# The Sperm Structure and Spermatogenesis of *Trypophloeus klimeschi* (Coleoptera: Curculionidae: Scolytinae)

**DOI:** 10.3390/biology10070583

**Published:** 2021-06-25

**Authors:** Jing Gao, Guanqun Gao, Jiaxing Wang, Hui Chen

**Affiliations:** 1 College of Forestry, Northwest A&F University, Yangling 712100, China; sxllgaojing@nwafu.edu.cn (J.G.); wjx2017@nwafu.edu.cn (J.W.); 2 Information Institute, Tianjin Academy of Agricultural Sciences, Tianjin 300192, China; ggqun@nwafu.edu.cn; 3 State Key Laboratory for Conservation and Utilization of Subtropical Agro-Bioresources, Guangdong Key Laboratory for Innovative Development and Utilization of Forest Plant Germplasm, College of Forestry and Landscape Architecture, South China Agricultural University, Guangzhou 510642, China

**Keywords:** male reproductive system, sperm ultrastructure, spermatogenesis, *Trypophloeus klimeschi* (Coleoptera: Curculionidae: Scolytinae)

## Abstract

**Simple Summary:**

In the mating, reproduction, and phylogenetic reconstruction of various insect taxa, the morphological characteristics of the male reproductive system, spermatogenesis, and sperm ultrastructure are important. We investigated these morphological characteristics of *Trypophloeus klimeschi* (Coleoptera: Curculionidae: Scolytinae), which is one of the most destructive pests of *Populus alba* var. *pyramidalis* (Bunge) using light microscopy, scanning electron microscopy, and transmission electron microscopy. We also compared these morphological characteristics with that found in other Curculionidae.

**Abstract:**

The male reproductive system, sperm structure, and spermatogenesis of *Trypophloeus* *klimeschi* (Coleoptera: Curculionidae: Scolytinae), which is one of the most destructive pests of *Populus alba* var. *pyramidalis* (Bunge), were investigated using light microscopy, scanning electron microscopy, and transmission electron microscopy. The male reproductive system of *T.* *klimeschi* is composed of testes, seminal vesicles, tubular accessory glands, multilobulated accessory glands, vasa deferentia, and a common ejaculatory duct. In spermatogenesis, two phenomena are apparent: The nuclear chromatin condenses into two different patterns, and an oval preacrosomal vesicle is present at the flank of the Golgi apparatus. The sperm are short, measuring 76.7 ± 1.8 μm in length, and are 508.1 ± 12.9 nm in width. The sperm are composed of a three-layer acrosomal complex, a cylindrical nucleus, two mitochondrial derivatives, a 9 + 9 + 2 axoneme, and two accessory bodies with a large “puff”-like expansion. Mature sperm are individually stored in seminal vesicles. During spermiogenesis, the similarities in the nuclear chromatin condensation characteristics of Curculioninae and Scolytinae are indicative of their close phylogenetic relationship. It appears that the preacrosomal vesicle being flanked by the Golgi apparatus is a characteristic of spermatogenesis in Curculionidae.

## 1. Introduction

Curculionidae, with about 48,000 valid species, is the largest family of known organisms [1]. Many pest species, such as *Scolytus schevyrewi*, *Scolytus seulensis*, *Scolytus rugulosus*, *Scolytus multistriatus*, *Trypophloeus populi*, *Lepyrus japonicuse*, *Sympiezomias velatus*, and *Cryptorhynchus lapathi*, attack poplar trees and other hardwood, causing significant economic and ecological problems worldwide [2,3,4,5,6]

*Trypophloeus klimeschi*, belonging to Scolytinae, Curculionidae [7], is one of the most destructive pests of *Populus alba* var. *pyramidalis* (Bunge). It was first recorded in the Kyrgyz Republic, which borders Xinjiang Uygur Autonomous Region in China [7,8,9]. Following an outbreak in the Dunhuang in recent years [2,10,11,12], this beetle has caused huge economic and ecological losses. *T. klimeschi* first invades branch shoots and then gradually spreads to the main trunk. The beetle bores and feeds in the phloem to form a gallery in which the eggs are deposited. The injured branches turn yellow and wither, and dense holes are formed in the trunk surface, causing the injured trees to wither and rapidly die [2]. The prevention and control of *T*. *klimeschi* must involve the control of population number [13]. Reproductive ability is one of the important factors that affect population number. Long-term field investigation has shown that *T*. *klimeschi* is a monogamous insect and mates once before laying eggs, and the number of eggs laid is as high as 30 [2]. Such a strong reproductive ability is the basis for its rapid spread. In order to better understand its mating and reproduction, morphological information of the male reproductive system, the sperm structure, and spermiogenesis is essential [14].

The morphological characteristics of the male reproductive system, spermatogenesis, and sperm ultrastructure have been considered important in taxonomy of various insect taxa [15,16,17,18,19,20,21,22,23]. The Scolytinae subfamily includes 6000 species belonging to 200 genera [24], and more studies of these areas are needed for its phylogenetic reconstruction considering the huge number of species. Only a few species of these areas have been studied, such as *Dendroctonus rnonticolae*, *Hypothenemus hampei*, *Dendroctonus armandi*, and *Hylurgus ligniperda* [13,25,26,27,28]. The male reproductive tract of most Scolytinae is composed of testes, seminal vesicles, accessory glands, vasa deferentia, and ejaculatory duct [13,25,26,27,28]. Their sperm usually contain a nucleus, a 9 + 9 + 2 axoneme, two accessory bodies, two mitochondrial derivatives, and one “puff”-like expansion [13,19]. However, there seem to be exceptions among these species. For example, *Hypothenemus hampei* has no accessory glands [26], and the sperm of *D. armandi* also contains a special large spongy body [13]. Although these studies revealed important information regarding the phylogenetic reconstruction of Scolytinae, these structures may vary significantly, even between closely related species [18]. There is still an urgent need for more research on the male reproductive system, sperm, and spermatogenesis of this subfamily.

Due to the wide variety and rapid spread of bark beetles, the lack of phylogenetic studies may lead to untimely identification and inadequate proliferation detection of their species. At present, only adult integument morphology, larval morphology, antenna receptor morphology, and life history can provide support for the systematic status of *T. klimeschi* [2,8,9]. Ning et al. predicts that *T. klimeschi* will quickly spread to more places in the future [29]. Therefore, the aim of this research was to study the reproductive system, spermatogenesis, and sperm ultrastructure of *T. klimeschi* using light microscopy, scanning electron microscopy, and transmission electron microscopy. This study provides key information for future research into mating, reproduction, and the systematic status of *T. klimeschi*, more evidence for phylogenetic reconstruction of Scolytinae, and potentially useful information for subsequent pest control.

## 2. Materials and Methods

### 2.1. Insects

*T. klimeschi* (larvae and pupae) collected from the bark of infested P. alba var. *pyramidalis* in Dunhuang City (40°06′50.61″ N, 94°36′10.24″ E), Gansu Province, China, were reared in 24-hole plates with feed containing *P. alba* var. *pyramidalis* bark powder in an artificial climate incubator (14L: 10D, 25 ± 1 °C, 65 ± 5% relative humidity) [2]. On the 1st, 8th, and 16th day after eclosion, 30 males were taken for later use in experiments. For anatomical analyses, the reproductive systems of 30 males—10 *T. klimeschi* each at 1, 8, and 16 days after eclosion—were observed with an OLYMPUS SZ2-ILST stereomicroscope and photographed with an OLYMPUS DP25 camera.

### 2.2. Scanning Electron Microscopy (SEM)

The reproductive systems of the 10 males in each age group were dissected and fixed in a solution containing 2.5% glutaraldehyde in 0.1 M phosphate (pH 7.2) for 12 h at 4 °C [2]. The samples were washed in phosphate-buffered saline (PBS), pH 7.2; dehydrated through a graded series of alcohol and isoamyl acetate; critical point dried with liquid CO2; and sputter coated with gold. Samples were then examined using a HITACHI S-4800 scanning electron microscope at 15 kV.

### 2.3. Transmission Electron Microscopy (TEM)

The 10 fixed samples of reproductive systems for each age group were rinsed with PBS, and post-fixation was performed in 1% osmium tetroxide for 1 h at 4 °C. After four 15 min washes in the same buffer, the samples were dehydrated through a graded ethanol series and embedded in 14381-UC LR WHITE. Semithin sections were obtained with a glass knife on a LEICA RM2265 microtome, stained with toluidine blue, and observed with an OLYMPUS BX43F microscope. Ultrathin sections (70 nm thick) were obtained with a diamond knife on an ultramicrotome (LEICA ULTRACUT UCT), routinely stained with uranyl acetate and lead citrate, and observed with a HITACHI HT7700 transmission electron microscope.

## 3. Results

### 3.1. Gross Morphology of the Male Reproductive System

**Figure 1:**
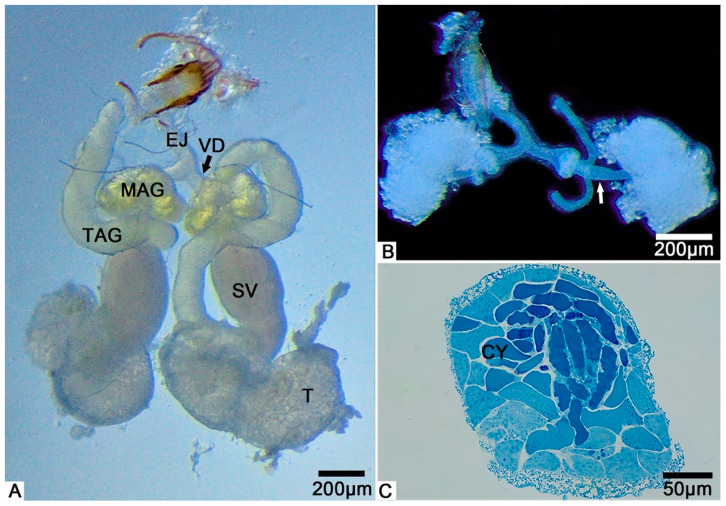
The male reproductive system of *T. klimeschi*: (**A**) The male reproductive system on the 16th day after eclosion. (**B**) On the first day after eclosion, the seminal vesicles are only thin tubules (white arrow). (**C**) The cross section of the testis showing many cysts (CY). Testis (T); seminal vesicle (SV); tubular accessory gland (TAG); multilobulated accessory gland (MAG); vas deferens (VD); ejaculatory duct (EJ).

From the light microscopy images, we observed that the male internal reproductive tract of *T. klimeschi* is composed of two units (Figure 1A). Each unit comprises a bilobed testis—a seminal vesicle inserted in the depression of the testis—two accessory glands (multilobulated gland and tubular gland), and the vas deferens (Figure 1A). The tubular accessory gland is connected to the seminal vesicle, while the multilobulated accessory gland surrounds the vas deferens (Figure 1A). Two units fuse at their posterior ends, flowing into an ejaculatory duct (Figure 1A). On the first day after eclosion, the seminal vesicles are only thin tubules (Figure 1B), which become thicker with maturation (Figure 1A).

**Figure 2:**
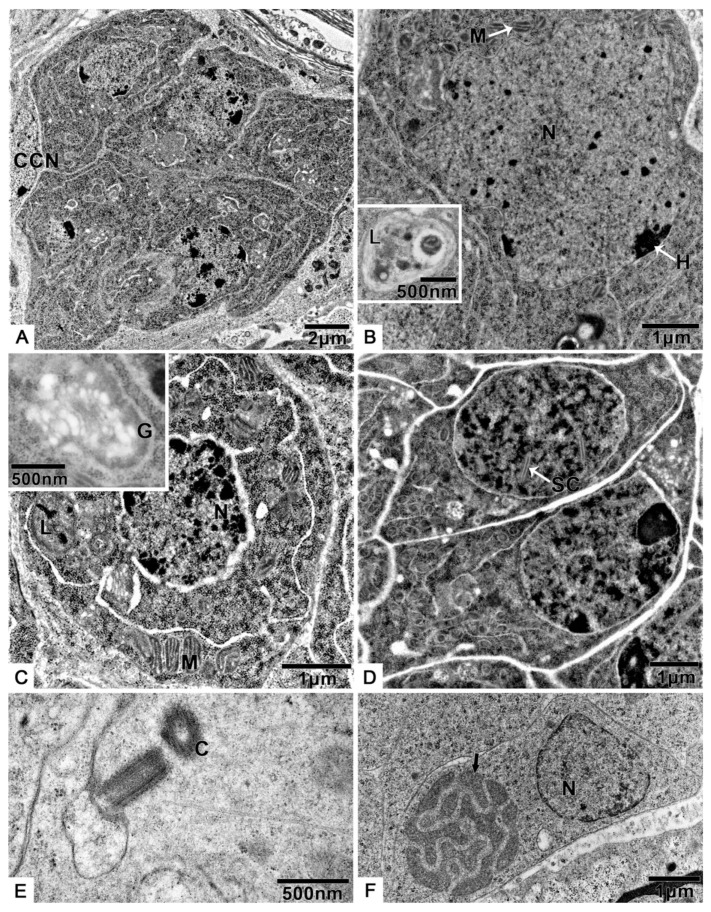
The spermatogenesis of *T. klimeschi*: (**A**) spermatogonia cyst; (**B**) spermatogonia; (**C**) primary spermatocytes; (**D**) secondary spermatocytes; (**E**) centrioles in spermatocytes; (**F**) spermatids. Cyst cell nucleus (CCN); centrioles (C); heterochromatin (H); mitochondria (M); Golgi apparatus (G); lysosomes (L); nucleus (N); synaptonemal complexes (SC); fusing mitochondria (black arrow).

The cysts were observed to completely fill the testes (Figure 1C). A cyst is a saccular structure composed of cyst cells (Figure 2A), within which spermatogenesis occurs. During spermatogenesis, spermatogonia undergo repeated mitotic divisions and give rise to spermatocytes. These spermatocytes undergo successive meiotic divisions and give rise to spermatids, which, after morphological change (spermiogenesis), form spermatozoa. In each cyst, 350–512 (n = 10) spermatozoa were observed, resulting from nine cell division cycles. Mature sperm were observed to fill the seminal vesicles.

### 3.2. Spermatogenesis

#### 3.2.1. From Spermatogonia to Spermatids

Transmission electron microscopy allowed us to determine that the spermatogonia are irregularly shaped cells (Figure 2A,B, Table S1), 5.6 ± 0.4 μm (n = 10) in diameter, with a large, irregularly shaped nucleus (3.5 ± 0.7 μm, n = 10). The nucleus contains homogeneously distributed granular chromatin and irregularly distributed electron-dense heterochromatin (Figure 2A,B). The cytoplasm contains many mitochondria and large lysosomes (Figure 2B).

The spermatocytes (Figure 2C, Table S1) originate from mitosis of spermatogonia and have a diameter of 4.8 ± 0.3 μm (n = 10). The cells are characterized by synaptonemal complexes in the round nucleus 3.1 ± 0.4 μm (n = 10) in diameter (Figure 2D, Table S1). Lysosomes, mitochondria with well-defined cristae, Golgi apparatus (Figure 2C), and two orthogonally arranged centrioles (Figure 2E) can be observed in the cytoplasm.

The spermatids (Figure 2F, Table S1) contain a round nucleus 2.1 ± 0.3 μm (n = 10) in diameter. Nuclear chromatin exhibits different degrees of compactness and electron density. This stage is characterized by the presence of fusing mitochondria (Figure 2F).

#### 3.2.2. Spermiogenesis

**Figure 3:**
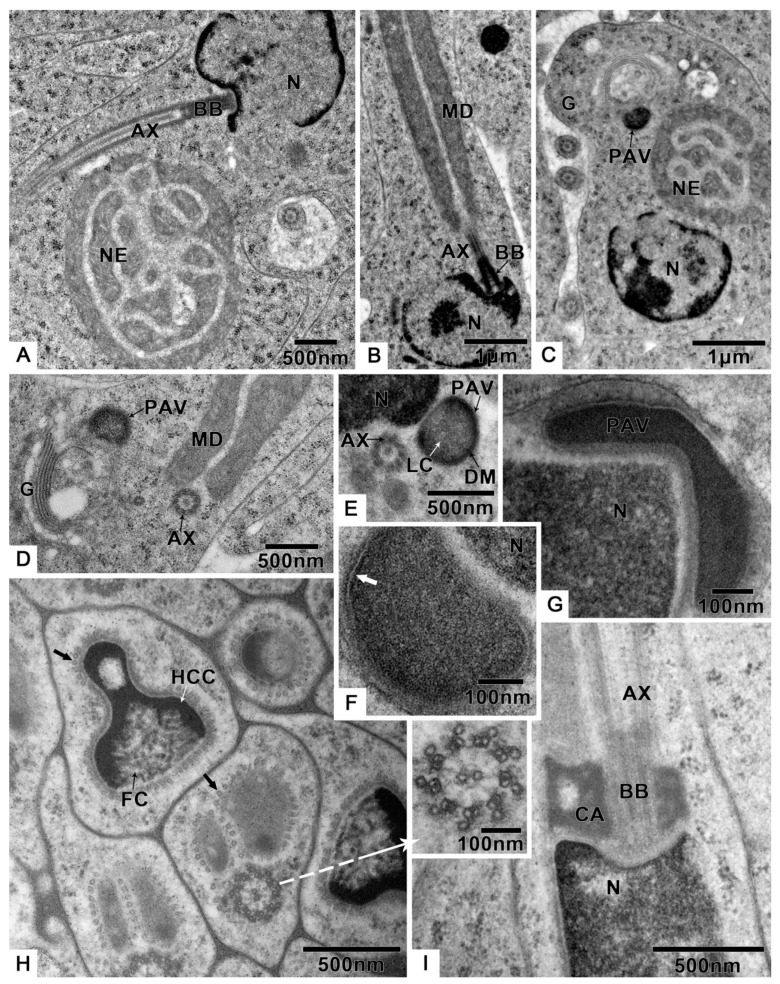
Spermiogenesis of *T. klimeschi*: (**A**) The axoneme starts elongating from the concavity of nucleus. (**B**) Mitochondrial derivatives are located on both sides of the axoneme and parallel to the axoneme. (**C**) Spermatid at initial stage of differentiation showing that the preacrosomal vesicle is near to the nebenkern and Golgi apparatus. (**D**,**E**) In the early spermatid (nebenkern becomes two mitochondrial derivatives), the preacrosomal vesicle and axoneme are near to the Golgi apparatus (**D**) and nucleus (**E**). The preacrosomal vesicle has a dark-stained mantle and light core (**E**). (**F**) The preacrosomal vesicle under high magnification and its membrane structure (white arrow). (**G**) As the nucleus elongates, the preacrosomal vesicle becomes flat. (**H**) The spermatid components are surrounded by microtubules (black arrow). The bell-shaped nucleus with different chromatin aggregation states: homogeneously compact chromatin and fibrillar chromatin. The cross section of the axoneme under high magnification (white arrow). (**I**) The centriolar adjunct appears in the spermatid stage. Nucleus (N); axoneme (AX); preacrosomal vesicle (PAV); nebenkern (NE); Golgi apparatus (G); dark-stained mantle (DM); light core (LC); homogeneously compact chromatin (HCC); fibrillar chromatin (FC); mitochondrial derivatives (MD); centriolar adjunct (CA); basal body (BB).

Germ cells undergo many changes during spermiogenesis: The nuclear chromatin becomes more concentrated, and the electron density is enhanced (Figure 3A,B). The lateroposterior end of the nucleus forms a concavity where the basal body is located, and the axoneme elongates from here (Figure 3A,B). A round preacrosomal vesicle, 448.3 ± 14.5 nm (n = 10) (Table S1) in diameter, is visible (Figure 3C). At the initial stage of spermatid differentiation, the preacrosomal vesicle is near the nebenkern and Golgi apparatus (Figure 3C). In the early spermatid, when the nebenkern becomes two mitochondrial derivatives, the preacrosomal vesicle is found near the Golgi apparatus (Figure 3D) and nucleus (Figure 3E). In more detail, the preacrosomal vesicle flanks and is near to the concave face of the Golgi apparatus (Figure 3D). In this stage, the preacrosomal vesicle has a dark-stained mantle and a light core (Figure 3E); both are surrounded by a membrane (Figure 3F). As the cell elongates, the preacrosomal vesicle flattens (Figure 3G). At a more mature stage, the bell-shaped nucleus is obvious, and its nuclear chromatin shows two distinct regions (Figure 3H): one homogeneously compact, the other fibrillar. At this stage of development, all sperm components are surrounded by microtubules (Figure 3H). Shortly after this, the centriolar adjunct is visible on both sides of the basal body (Figure 3I). However, there are no centriolar adjuncts in the mature sperm.

**Figure 4:**
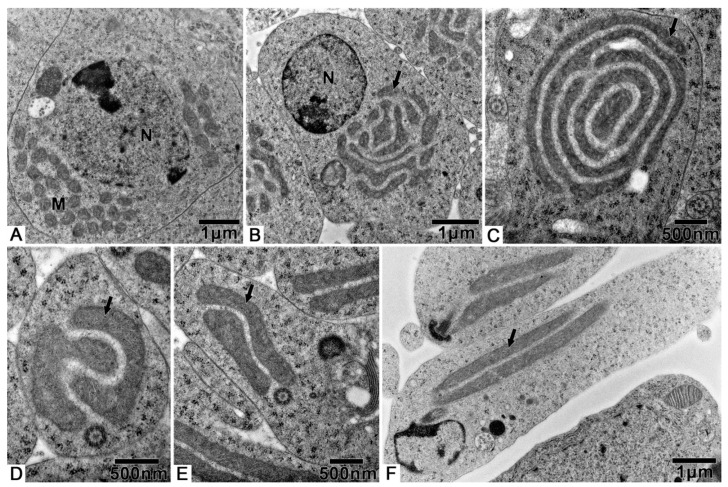
Changes of mitochondria during spermiogenesis of *T. klimeschi*: (**A**) Mitochondria (M) gathered together; (**B**–**F**) changing mitochondria (black arrow). Nucleus (N).

During spermiogenesis, the spherical-shaped mitochondria, which are found dispersed in the cytoplasm, begin to gather (Figure 4A). Then, the formation of two mitochondrial derivatives begins via stages that are twinned (Figure 4B), five-layer (Figure 4C) and two-layer nebenkern (Figure 4D,E). These two mitochondrial derivatives become slender (Figure 4F) as the spermatid and axoneme elongate (Figure 3B).

### 3.3. Spermatozoa

**Figure 5:**
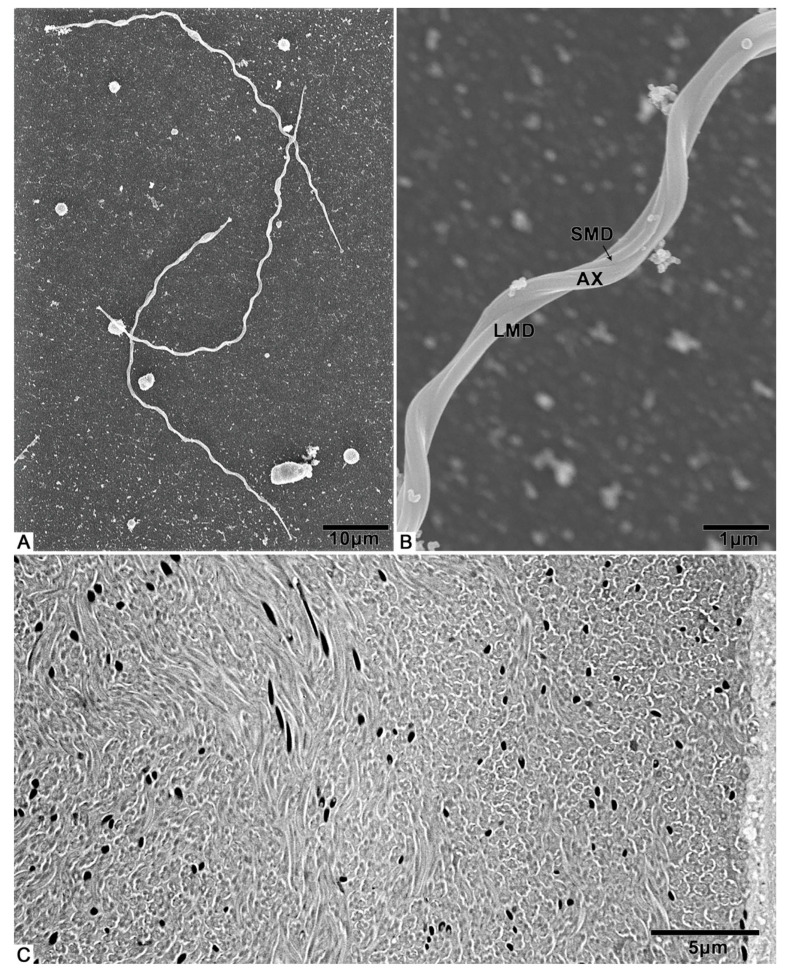
SEM and TEM micrographs of *T. klimeschi* spermatozoa: (**A**) The appearance of spermatozoa; (**B**) Detailed view of spermatozoa; (**C**) TEM micrograph of spermatozoa in seminal vesicles. The black, round, and short strips are the sperm nuclei. Axoneme (AX); large mitochondrial derivative (LMD); small mitochondrial derivative (SMD).

**Figure 6:**
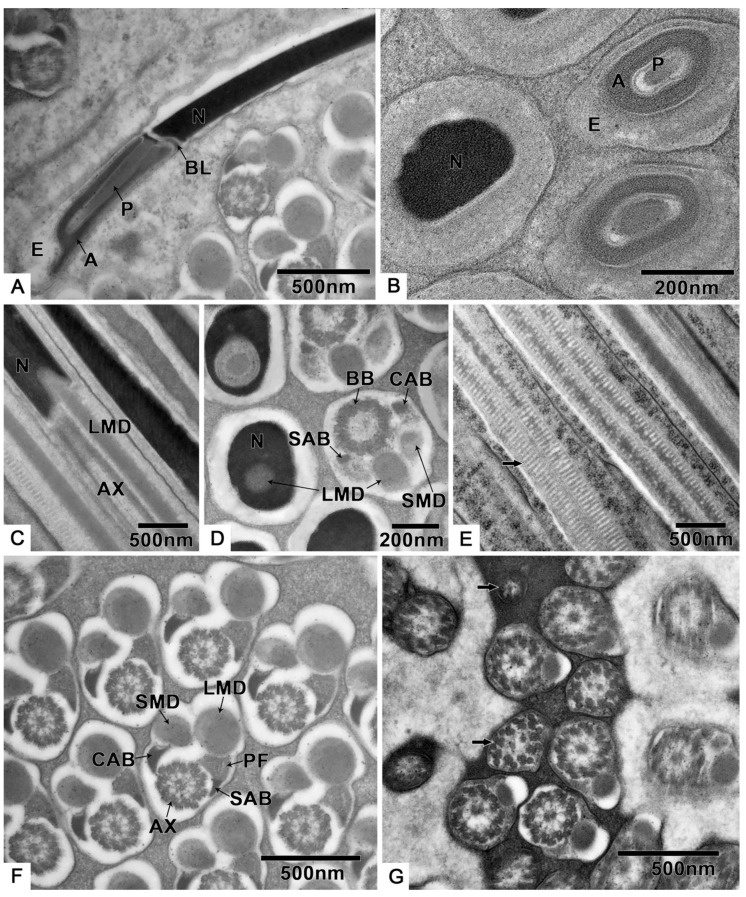
The spermatozoa structure of *T. klimeschi*: (**A**,**B**) The longitudinal and cross sections of 3-layered acrosome complex. (**C**,**D**) The longitudinal and cross sections showing the junction of the nucleus and flagellar components. (**E**) The mitochondrial cristae (black arrow). (**F**) Cross section of the middle of the spermatozoa. (**G**) Cross section showing the disorganized axoneme (black arrow) at the end of the spermatozoa. Extra acrosomal layer (E); acrosomal vesicle (A); perforatorium (P); nucleus (N); basal lamina (BL); axoneme (AX); large mitochondrial derivative (LMD); small mitochondrial derivative (SMD); crescent accessory body (CAB); basal body (BB); small accessory body (SAB); puff-like expansion (PF).

The spermatozoa of *T. klimeschi* are slender (Figure 5A, Table S1), measuring 76.7 ± 1.8 μm (n = 10) in length and 508.1 ± 13.0 nm (n = 10) in width, and composed of an acrosomal complex, a nucleus (Figure 6A), an axoneme, two mitochondrial derivatives, and two accessory bodies (Figure 6F). The mitochondrial derivatives do not embrace the axoneme but rather run approximately parallel to it (Figure 5B). Mature sperm are stored in the seminal vesicles and its cross section, showing that the sperm of *T. klimeschi* are arranged very closely on the 16th day after eclosion (Figure 5C).

The three-layer acrosomal complex (Figure 6A,B, Table S1), measuring 1.2 ± 0.0 μm (n = 10) in length, is made up of a dense extra acrosomal granular layer, a cup-like acrosomal vesicle, and a rod-like conical perforatorium. The acrosomal complex lies on a slightly concave nuclear face and is separated from the nucleus by a thick basal lamina (Figure 6A).

The nucleus is close to the acrosomal complex (Figure 6A,B) with a circular cross section, which is indicated by a longitudinal section that is 6.0 ± 0.2 μm (n = 10) in length and 289.9 ± 22.6 nm (n = 10) in diameter (Table S1).

The longitudinal and cross sections (Figure 6C,D) show that the flagella components are inserted into the nucleus, while the large mitochondrial derivative is the deepest. The 9 + 9 + 2 axoneme (Figure 6F, Table S1) has a diameter of 282.9 ± 8.1 nm (n = 10). The large mitochondrial derivative with distinct cristae (Figure 6E), 236.0 ± 18.9 nm (n = 10) in diameter is always thinner than the axoneme and becomes thinner along the flagellum before completely disappearing (Figure 6G).

The two accessory bodies are different in shape (Figure 6F). From the cross section of the middle part of the sperm, we know that the crescent accessory body is smaller than one-quarter of the axoneme size, and the other is small and mostly triangular (sometimes crescent-shaped) with a large, compact, “puff”-like expansion.

At the end of the sperm, the two accessory bodies are the first to disappear, followed by the smaller mitochondrial derivative, larger mitochondrial derivative, and, finally, the disorganized disappearance of the axoneme (Figure 6G).

## 4. Discussion

In terms of general morphology, the male reproductive tract of *T. klimeschi* is composed of two testes, two seminal vesicles, two tubular accessory glands, two multilobulated accessory glands, two vasa deferentia, and an ejaculatory duct, and this composition is identical to that reported for other Curculionidae [13,25,26,27,30,31,32].

*T. klimeschi* has two types of accessory gland. The multilobulated accessory glands of some Scolytinae—for instance, *D. armandi* [13], *Scolytus destructor* [28], *H. ligniperda* [27], and *D. rnonticolae* [25]—correspond to the prostate glands of some species of other Curculionidae subfamilies, such as *Hyperodes bonariensis* (Coleoptera: Curculionidae: Cylindrorhininae) [31], *Listronotus bonariensis* (Coleoptera: Curculionidae: Cylindrorhininae) [33], and *Tanymecus dilaticollis* (Coleoptera: Curculionidae: Entiminae) [32]. Because the two structures have a similar appearance (they are both circular and multilobulated) and position (they are both next to tubular accessory glands and surround vasa deferentia), it is possible that they have the same structure. However, *H. hampei* (Coleoptera: Curculionidae: Scolytinae) [26] does not have this accessory gland.

In *T. klimeschi*, spermatogenesis occurs in cysts, as is the case for most insects [34,35,36,37,38]. The number of sperm per cyst is less than 512 as a result of nine cycles (2^9^) of cell division. In Coleoptera, the number of divisions is between 4 to 10 [13,21,34,39]. According to the hypothesis of Virkki [40], insects of more archaic orders have more sperm cells per bundle than their more modern counterparts; if so, *T. klimeschi* may be a primitive coleopteran species.

It seems that two different patterns of nuclear chromatin condensation during spermiogenesis (one showing the chromatin more homogeneously condensed and another showing the chromatin with a fibrillar aspect) is a shared feature of Curculionidae [13,21,30,34,41]. However, the process of condensation is different and can generally be divided into two types: one having a honeycomb chromatin stage, such as for *Sitophilus zeamais* (Coleoptera: Curculionidae: Dryophthorinae), *Sitophilus oryzae* (Coleoptera: Curculionidae: Dryophthorinae) [21], and *Rhynchophorus ferrugineus* (Coleoptera: Curculionidae: Rhynchophorinae) [41], and the other not, such as for *Anthonomus grandis* (Coleoptera: Curculionidae: Curculioninae) [30], and *D. armandi* [13]. *T. klimeschi* belongs to the latter. In addition, another feature of the latter is its bell-shaped nucleus [13,34]. This result supports the theory that there is a close relationship between Curculioninae and Scolytinae, and Rhynchophorinae and Dryophthorinae [19,42].

In insects, as in animals in general, the acrosome is a product of the Golgi apparatus [43]. The position of the acrosome changes as the sperm matures. However, in the early spermatid, for many insects, the preacrosomal vesicle is situated on the concave face of the Golgi apparatus, between its innermost cisternae and the spermatid nucleus, such as in *Euschistus* (Hemiptera: Pentatomidae) [43], some Orthoptera [44], *Acheta domesticus* (Orthopteroidea, Ensifera) [45], *Sciara coprophila* (Diptera, Sciaridae) [46,47], and *Machilontus* sp. (Archaeognatha: Meinertellidae) [48]. However, in *Conocephalus* (Orthoptera: Tettigoniidae) [43], the preacrosomal vesicle is situated on the convex side of the Golgi apparatus, which is situated between the preacrosomal vesicle and the nucleus. In this study, the preacrosomal vesicle is situated on the flank of the Golgi apparatus and near to the concave face of the Golgi apparatus. The preacrosomal vesicle was also found in the same position in some Curculionidae, for example, *S. oryzae* [21] and *R. ferrugineus* [49]. However, unlike the bean-shaped preacrosomal vesicle of *S. oryzae* and the round preacrosomal vesicle of *R. ferrugineus*, the vesicle of *T. klimeschi* is oval-shaped. This special position of the preacrosomal vesicle may be a characteristic of Curculionidae.

The spermatozoa of *T. klimeschi* are linear and slender and are similar to the general description for other Curculionidae sperm [13,21,30,50]. Compared to spermatozoa of other Curculionidae, which are 110 to 300 μm in length [19], they are short, with a length of about 77 μm, though they share a similar structure to most [13,19,21,22,30,50,51]: a three-layer acrosome with a cup-like acrosome vesicle; a 9 + 9 + 2 axoneme; two mitochondrial derivatives of different sizes; a large mitochondrial derivative, which is always thinner than the axoneme in, for example, *D. armandi* [13], *Kissophagus hederae* (Coleoptera: Curculionidae: Scolytinae), and *Ernoporus fagi* (Coleoptera: Curculionidae: Scolytinae) [19]; two accessory bodies; and one large, compact, “puff”-like expansion.

At present, two types of insect spermatozoa storage have been characterized: in bundles and individually [18,52,53,54,55]. Bundled spermatozoa are maintained in primary arrangement (the arrangement in the testes), and their heads are inserted into the extracellular matrix. However, there seems to be no description of how spermatozoa are individually stored. In this study, according to the cross section of the seminal vesicle of *T. klimeschi* (Figure 5C), we determined that the sperm are arranged very tightly, but the sperm heads are not always gathered together as is the case for the heads of sperm stored in bundles [55]. Instead, the sperm heads are dispersed similarly to when they are stored individually [53]. Therefore, based on this observation, we believe that the sperm of *T. klimeschi* are individually stored in seminal vesicles.

## 5. Conclusions

In conclusion, the general morphology of the male reproductive tract, spermatogenesis, and the structure of the spermatozoa of *T. klimeschi* is, for the most part, similar to that of the majority of the Scolytinae. However, the spermatozoa of *T. klimeschi* is, at approximately 77 μm, very short. During spermiogenesis, the similarity in the nuclear chromatin condensation characteristics of Scolytinae and Curculioninae is indicative of their close phylogenetic relationship. The preacrosomal vesicle flanking the Golgi apparatus seems to be a characteristic of spermatogenesis in Curculionidae. This study provides novel information on the reproductive biology of the Curculionidae family and develops the existing taxonomic and phylogenetic research on Curculionidae.

## Data Availability

Not applicable.

## Supplementary Materials

The following are available online at https://www.mdpi.com/article/10.3390/biology10070583/s1. Table S1: Size of different cells and organelles.
